# The Comparison of Fresh and Dry Duckweed (*Lemna minor* L.) on Metal (Cr^6+^, Cd^2+^, and Zn^2+^) Removal from Wastewater

**DOI:** 10.3390/plants15050848

**Published:** 2026-03-09

**Authors:** Rahin Islam, Noah Smith, Ben Jang, Lin Guo

**Affiliations:** 1 Department of Biological and Environmental Sciences, East Texas A&M University, Commerce, TX 75428, USA; rislam3@leomail.tamuc.edu; 2 Department of Chemistry, East Texas A&M University, Commerce, TX 75428, USA; nsmith23@leomail.tamuc.edu (N.S.); ben.jang@etamu.edu (B.J.)

**Keywords:** duckweed, metals, biosorption, phytoremediation, Cr^6+^, Cd^2+^, Zn^2+^

## Abstract

Heavy metals contaminating the environment is a global concern. Duckweed (*Lemna minor*) is a promising plant for the phytoremediation and biosorption of metal-contaminated water. Although studies have shown that duckweed can remove multiple metals, there is limited research comparing the efficiency of fresh and dried biomass for wastewater treatment. To evaluate the performance of both forms, fresh and dried duckweed were exposed to metal solutions containing varying concentrations of Cr^6+^, Cd^2+^, and Zn^2+^ (5 mg/L Cr^6+^ + 1 mg/L Cd^2+^ 10 mg/L Zn^2+^; 10 mg/L Cr^6+^ + 5 mg/L Cd^2+^ + 50 mg/L Zn^2+^; or 50 mg/L Cr^6+^ + 25 mg/L Cd^2+^ + 250 mg/L Zn^2+^) for a duration of 168 h. Metal uptake in fresh duckweed followed zero-order kinetics for Cr^6+^, Cd^2+^, and Zn^2+^ sequestration or Michaelis–Menten kinetics for Cd^2+^ and Zn^2+^ uptake, rather than a first-order model. In contrast, dried duckweed reached equilibrium more rapidly, within 4–48 h, exhibiting pseudo-second-order kinetic and fitting the Langmuir isotherm model. Zn^2+^ reached equilibrium the fastest (4 h), Cd^2+^ required 4–24 h, and Cr^6+^ required up to 48 h to reach equilibrium. In general, fresh duckweed uptakes more metals over the 168 h period, depending on the metal type and concentration. However, dried duckweed demonstrated a rapid remediation capability. The findings highlight the complementary potential of applying both fresh and dried duckweed for wastewater treatment.

## 1. Introduction

While some metals occur naturally in the environment, human activities have significantly increased their concentrations, raising global concern about their persistent detrimental effects on ecosystems and human health [1]. The biological impact of metals varies considerably. Some, like zinc, are essential micronutrients; however, consumption above recommended levels can cause adverse effects such as anemia and neutropenia [2,3]. Conversely, non-essential metals like cadmium are toxic even at minimal doses and can lead to adverse effects such as skeletal demineralization and liver damage [2,3]. Chromium is a naturally occurring metal but can also be released by industrial processes. It exists in several oxidation states, such as trivalent (Cr^3+^) and hexavalent (Cr^6+^) [4]. Among these, Cr^6+^ is markedly more toxic than other forms and poses severe hazards to living organisms [5]. Exposure to Cr^6+^ has been linked to a range of health issues, from cancer to allergic dermatitis, in humans and wildlife [5].

Traditional methods for removing metals from environments—including chemical precipitation, coagulation, and ion-exchange—can cause secondary environmental problems [6,7]. Consequently, more cost-effective and environmentally friendly biological alternatives have gained attention. These include phytoremediation, which uses plants to cleanse environmental pollutants [8] and biosorption, which utilizes biological materials such as algae, bacteria, and fungi to sequester contaminants [9,10,11].

*Lemna minor* L. (duckweed), in both fresh and dry forms, is considered an effective agent for phytoremediation and biosorption. Its fast growth rate and high biomass production enable the removal of significant amounts of contaminants from municipal and industrial wastewater [12,13,14]. For example, several studies reported high removal efficiencies for Cr^6+^ using fresh *L. minor* [15]. Living duckweed has also been shown to actively uptake metals such as Cd^2+^, Pb^2+^, and Zn^2+^ through intracellular sequestration [16]. However, the performance of living duckweed may vary depending on the type and toxicity of metals [12]. In parallel, increasing attention has been given to dried *L. minor* biomass as an efficient biosorbent. Recent research also indicated that dry duckweed exhibited strong affinity for metal ions, including Cr^6+^, Cu^2+^, and Cd^2+^ [17,18]. Unlike fresh plants, dry biomass is not affected by metal toxicity, improving its practicality for industrial wastewater treatment [19].

Despite the recognized potential of *L. minor* for metal removal, comparative studies systematically evaluating the performance of fresh versus dry duckweed under identical experimental conditions remain limited. Most existing studies focus exclusively on either living plants (phytoremediation) or dried biomass (biosorption), often treating these approaches independently. As a result, critical knowledge gaps persist regarding differences in removal efficiency, tolerance to metal toxicity, and underlying mechanisms between fresh and dry forms—particularly in wastewater containing complex metal mixtures.

In addition, distinct kinetic behaviors have been reported for metal removal by fresh (living) and dry (non-living) *L. minor*, reflecting fundamental differences in their uptake mechanisms. While fresh duckweed often exhibits multi-phase or metabolism-dependent kinetics [15,20,21], dry duckweed is typically described by pseudo-second-order or diffusion-controlled models [19]. However, most studies focus on only one biomass state. The uptake kinetics for fresh and dry duckweed remain poorly resolved due to limited comparative studies and inconsistent modeling approaches [22,23].

Therefore, this study provided a side-by-side, mechanistic, and performance-based evaluation of the two most common duckweed forms under multi-metal conditions. The specific objectives were to: investigate the capacity and kinetics of Zn^2+^, Cd^2+^, and Cr^6+^ removal by both fresh and dry *L*. *minor* from wastewater; and determine the adsorption isotherms of Zn^2+^, Cd^2+^, and Cr^6+^ for the dry duckweed biomass. This research can provide new insights into the relative effectiveness of metabolic uptake versus passive biosorption, the influence of biomass state on metal removal pathways, and practical implications for wastewater treatment design. Additionally, it can contribute to a more integrated understanding of duckweed-based remediation strategies and support the development of optimized and sustainable technologies for treating metal-contaminated waters.

## 2. Results

### 2.1. Phytoremediation by Fresh Duckweed

**Figure 1:**
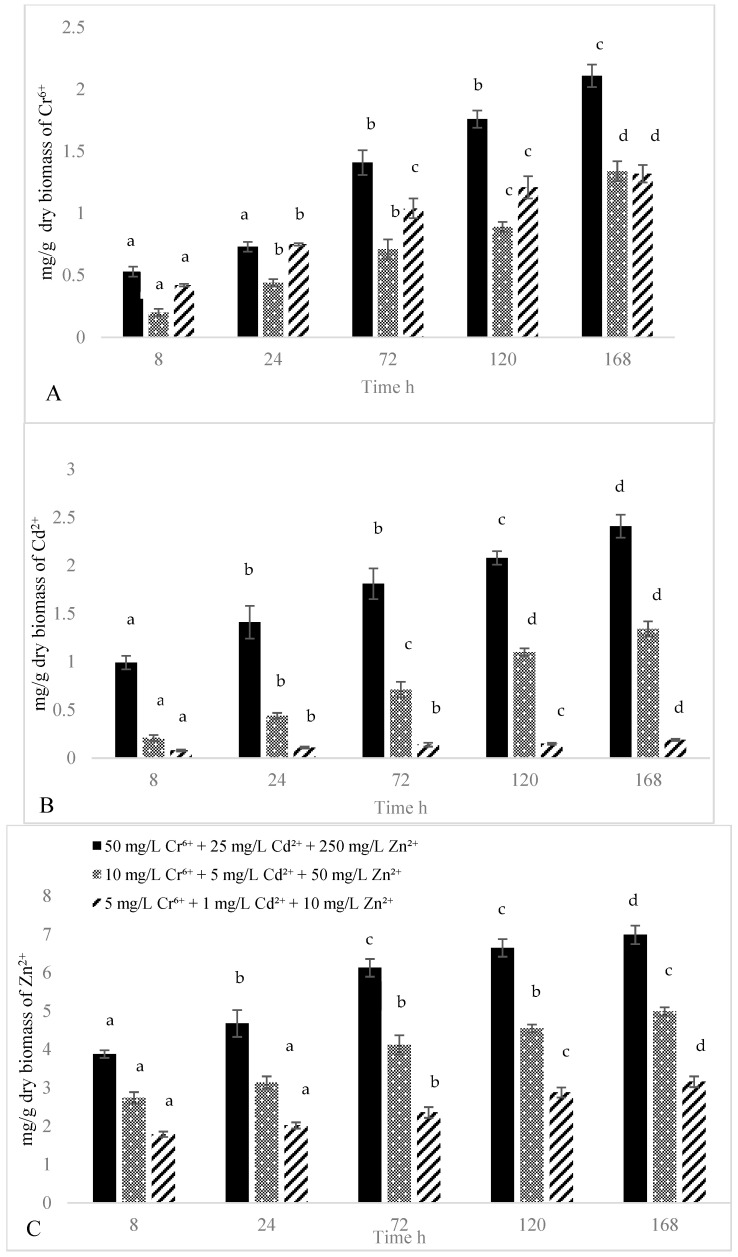
The amounts of Cd^2+^ (**A**), Cr^6+^ (**B**) and Zn^2+^ (**C**) accumulated in fresh duckweed (mg/g dry biomass) cultured in solutions with different levels of metals. Different letters indicated a significant difference at *p* < 0.05. n = 4.

Yellowing was observed in duckweed exposed to medium and high metal concentrations. However, all duckweed survived because their root systems remained attached to the fronds. No significant differences in biomass growth were found between the treatment groups and the control. However, the accumulation of metals in fresh duckweed increased over the 168 h period. More Zn^2+^ was sequestered in duckweed compared to Cd^2+^ and Cr^6+^. For example, the duckweed accumulated 0.53 ± 0.04 mg/g of dry biomass of Cr^6+^ after 8 h while the amounts significantly (*p* < 0.05) increased to 1.76 ± 0.07 mg/g of dry biomass after 120 h (Figure 1A). After 8 h, the Cd^2+^ concentration in duckweed cultured in the middle level of metals (10 mg/L Cr^6+^ + 5 mg/L Cd^2+^ + 50 mg/L Zn^2+^) was 0.2 ± 0.03 mg/g dry biomass, which subsequently increased to 1.34 ± 0.08 mg/g dry biomass after 168 h (*p* < 0.05) (Figure 1B). The Zn^2+^ concentration in duckweed cultured in a low level of metals (5 mg/L Cr^6+^ + 1 mg/L Cd^2+^ + 10 mg/L Zn^2+^) also significantly increased from 1.79 ± 0.07 mg/g dry biomass at 8 h to 3.16 ± 0.14 mg/g dry biomass at 168 h (Figure 1C). In general, more metals were sequestered in duckweed cultured in solutions with high levels of metals, compared to those plants that grew in low or middle levels. For example, duckweed cultured in high level of metal solutions (50 mg/L Cr^6+^ + 25 mg/L Cd^2+^ + 250 mg/L Zn^2+^) accumulated 1.81 ± 0.16 mg/g dry biomass of Cd^2+^ and 6.13 ± 0.23 mg/g dry biomass of Zn^2+^, which were significantly (*p* < 0.05) higher than those grew in solutions with low levels of metals (0.14 ± 0.02 mg/g dry biomass of Cd^2+^ and 2.36 ± 0.14 mg/g dry biomass of Zn^2+^) after 72 h (Figure 1).

**Figure 2:**
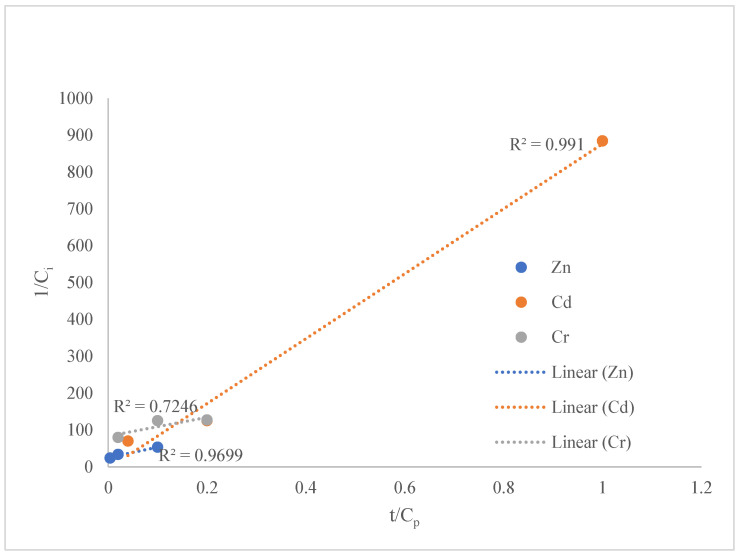
Linearized Michaelis–Menten model fitting for Zn^2+^, Cd^2+^ and Cr^6+^ at 168 h.

According to kinetic analysis, metal sequestration in fresh duckweed fit better to a zero-order model than a first-order model (Table 1). The fit to zero-order kinetics indicated that metal removal by fresh duckweed increased at a near-constant rate over the first 168 h. By plotting t/C_p_ against 1/C_i_, the linearized Michaelis–Menten model produces a straight line [24]. Figure 2 illustrates that the Michaelis–Menten model fit Cd^2+^ and Zn^2+^ well at 168 h. As shown in Table 2, the model also exhibited very high R^2^ values for Cd^2+^ and Zn^2+^ uptake at other exposure times in duckweed.

### 2.2. Biosorption by Dry Duckweed

**Figure 3:**
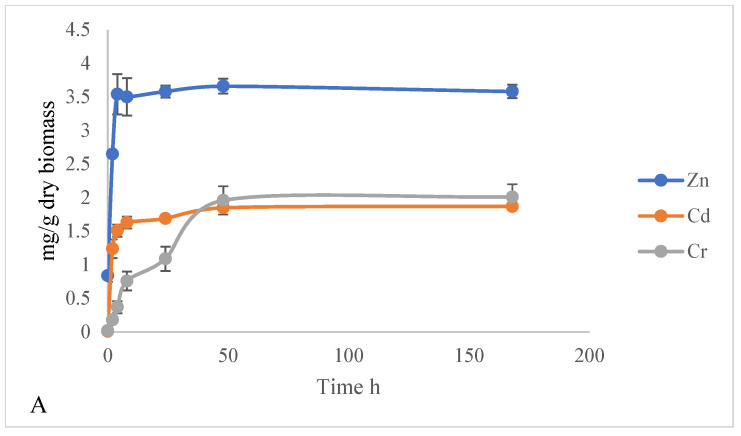

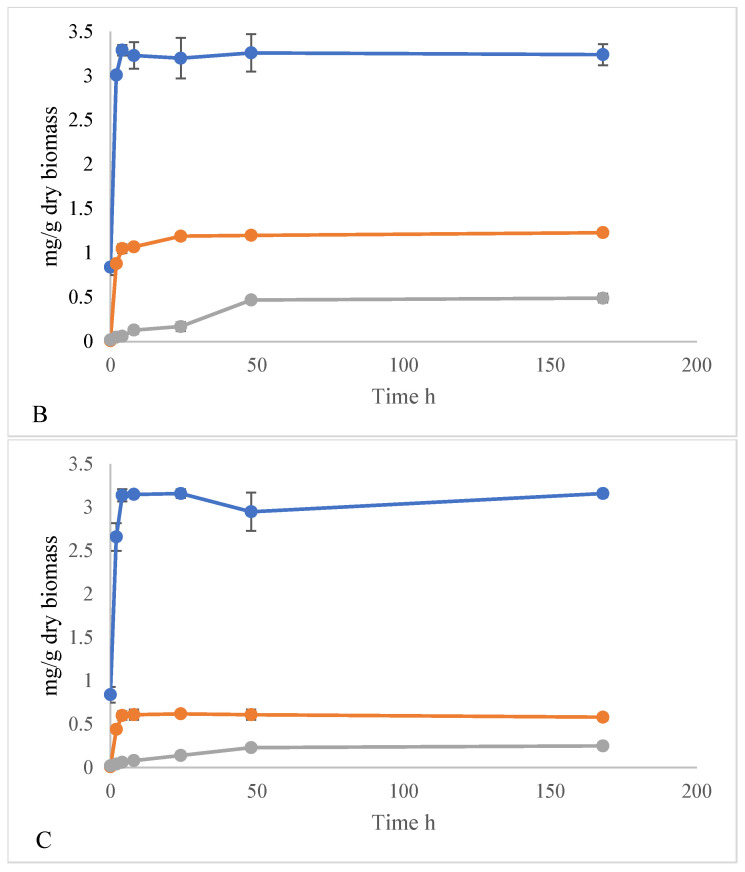
Metals adsorbed to dry duckweed (mg/g dry biomass) cultured in solutions with 50 mg/L Cr^6+^ + 25 mg/L Cd^2+^ + 250 mg/L Zn^2+^ (**A**), 10 mg/L Cr^6+^ + 5 mg/L Cd^2+^ + 50 mg/L Zn^2+^ (**B**), or 5 mg/L Cr^6+^ + 1 mg/L Cd^2+^ + 10 mg/L Zn^2+^ (**C**). Zn^2+^ reached equilibrium the fastest (4 h), Cd^2+^ required 4–24 h, and Cr^6+^ required up to 48 h to reach equilibrium.

Dry duckweed exhibited quick adsorption, but the time to achieve equilibrium under different metal conditions were different. For example, dry duckweed exhibited quick adsorption in the first 48 h under high Cr^6+^ conditions, achieving equilibrium at about 2.0 mg/g dry biomass (Figure 3A, Table 2). Adsorption of Cr^6+^ onto dried duckweed under medium-metal conditions (10 mg/L Cr^6+^ + 5 mg/L Cd^2+^ + 50 mg/L Zn^2+^) increased dramatically during the initial 48 h, reaching an equilibrium of approximately 0.5 mg/g dry biomass (Figure 3B, Table 2). Under low-metal conditions (5 mg/L Cr^6+^ + 1 mg/L Cd^2+^ + 10 mg/L Zn^2+^), dried duckweed adsorbed Cr^6+^ rapidly within the first 48 h, reaching an equilibrium concentration of approximately 0.25 mg/g dry biomass (Figure 3C, Table 2). This value was lower than the equilibrium levels observed under medium- and high-level conditions.

Under high-metal conditions (50 mg/L Cr^6+^ + 25 mg/L Cd^2+^ + 250 mg/L Zn^2+^), dried duckweed reached an equilibrium concentration of approximately 1.8 mg/g dry biomass of Cd^2+^ within 48 h (Figure 3A, Table 2). No significant further adsorption was observed thereafter. Under medium-metal conditions (10 mg/L Cr^6+^ + 5 mg/L Cd^2+^ + 50 mg/L Zn^2+^), the adsorption of Cd^2+^ onto dried duckweed increased dramatically within 24 h, instead of 48 h (Figure 3B). The process reached equilibrium at an adsorption capacity of approximately 1.2 mg/g dry biomass of Cd^2+^ (Figure 3B, Table 2). Compared to medium and high levels, dried duckweed attained adsorption equilibrium much faster under low metal conditions (5 mg/L Cr^6+^ + 1 mg/L Cd^2+^ + 10 mg/L Zn^2+^), reaching a capacity of 0.65 mg/g dry biomass of Cd^2+^ within 4 h (Figure 3C, Table 2). The rapid kinetics indicated immediate saturation of the available surface-binding sites.

Compared to Cr^6+^ and Cd^2+^, Zn^2+^ adsorption on dried duckweed at high-metal concentrations (50 mg/L Cr^6+^ + 25 mg/L Cd^2+^ + 250 mg/L Zn^2+^) was exceptionally rapid, reaching an equilibrium of approximately 3.8 mg/g dry biomass of Zn^2+^ within just 4 h (Figure 3A, Table 2). Zn^2+^ also reached a higher equilibrium concentration than either Cr^6+^ or Cd^2+^. Zn^2+^ adsorption on dried duckweed at medium-metal concentrations (10 mg/L Cr^6+^ + 5 mg/L Cd^2+^ + 50 mg/L Zn^2+^) was also rapid, reaching equilibrium within 4 h at approximately 3.4 mg/g dry biomass (Figure 3B, Table 2). The adsorption of zinc onto dried duckweed under low-metal conditions (5 mg/L Cr^6+^ + 1 mg/L Cd^2+^ + 10 mg/L Zn^2+^) also featured a rapid initial phase, followed by stabilization at an equilibrium capacity of 3.0 mg/g dry biomass after 4 h (Figure 3C, Table 2).

As shown in Table 2, the high R^2^ values for the second-order model versus those for the first-order model indicated it much better described the adsorption kinetics of Cr^6+^, Cd^2+^ and Zn^2+^ in dried duckweed. This suggested that the process was dominated by chemisorption, which involved the sharing or exchange of electrons between metal ions and functional groups [25,26]. Overall, the consistently high second-order fits implied that chemical bonding and reactive site availability, rather than straightforward concentration gradients, regulated uptake [25,27].

The pseudo-first-order rate constant k_1_ and the corresponding equilibrium capacity Q_e1_, along with the pseudo-second-order rate constant k_2_ and its equilibrium capacity Q_e2_, are also presented in Table 2. It was evident that the discrepancy between Q_e1_ and the experimental equilibrium capacity Q_e-exp_ was larger, whereas the Q_e2_ values were closer to the experimental results. This further supported that the pseudo-second-order model better fits the adsorption data for duckweed. In this study, the pseudo-second-order rate constant k_2_ was higher for Zn^2+^ than for Cd^2+^, and notably higher than for Cr^6+^. Previous research also reported that adsorption rate constants for Cd^2+^ and Zn^2+^ typically ranged from approximately 0.6 to 12 g/(mg.h), while Cr^6+^ generally exhibited a lower k_2_ value under similar [28]. In addition, the pseudo-second-order constant is influenced by the type and concentration of metals, with a higher pseudo-second-order rate constant generally indicating faster sorption kinetics [29,30]. The higher k_2_ values observed for Zn^2+^ and Cd^2+^ compared to Cr^6+^ further supported our finding that Zn^2+^ reached equilibrium the fastest (within 4 h), Cd^2+^ required between 4 and 24 h, and Cr^6+^ took up to 48 h to reach equilibrium.

The adsorption of metals onto duckweed is typically described by two primary isotherm models. The Langmuir model assumes monolayer adsorption onto a surface with homogeneous binding sites, yielding a maximum adsorption capacity (Q_m_) often associated with chemisorption [25,26]. In contrast, the Freundlich model is an empirical equation that describes multilayer adsorption on heterogeneous surfaces, with constants representing adsorption capacity and intensity [25,27]. The adsorption of Cr^6+^, Cd^2+^ and Zn^2+^ onto duckweed was best described by the Langmuir isotherm model (R^2^ = 0.99–1.00) (Table 3), indicating monolayer adsorption onto a homogeneous surface with equivalent binding sites. Conversely, the correlations in the Freundlich model were lower, particularly for Zn^2+^. The Q_m_ was also predicted using the Langmuir isotherm model (Table 3). The adsorption of Zn^2+^ under high-concentration conditions in our experiment nearly reached the maximum capacity (3.90 mg/g dry biomass of Zn^2+^).

K_L_ is the Langmuir constant (L/mg), which reflects the affinity between metal ions and binding sites and indicates the strength of binding between the adsorbents and the metals [31] is also presented in Table 3. The K_L_ values obtained in our study were consistent with those reported in previous research. For instance, K_L_ values ranging from 0.01 to 0.05 for Cd^2+^ and 0.02 to 0.08 for Cr^6+^ were reported in dried duckweed (*Wolffia globosa*) [32] Similarly, a K_L_ of 0.32 for Zn^2+^ was found in *Spirulina platensis* [33], while values of 0.006 for Cd^2+^ in *Spirodela intermedia*, 0.014 for Cd^2+^ in *L. minor*, and 0.002 for Cd^2+^ in *Pistia stratiotes* were documented [26]. A higher K_L_ of 0.79 for Cd^2+^ was also reported in *Caladium bicolor* (wild cocoyam) biomass [34]. The Langmuir constant reflects the affinity between adsorbents and metals and is also influenced by the specific adsorbent material and experimental conditions such as temperature, pH, and metal concentrations [34,35]. In our study, the K_L_ values for Zn^2+^ and Cd^2+^ were higher than that for Cr^6+^, indicating that dried duckweed has a greater affinity for Zn^2+^ and Cd^2+^ than for Cr^6+^. This finding further supported our observation that Zn^2+^ and Cd^2+^ were adsorbed more readily and rapidly than Cr^6+^.

### 2.3. Comparison of Fresh and Dry Duckweed

**Figure 4:**
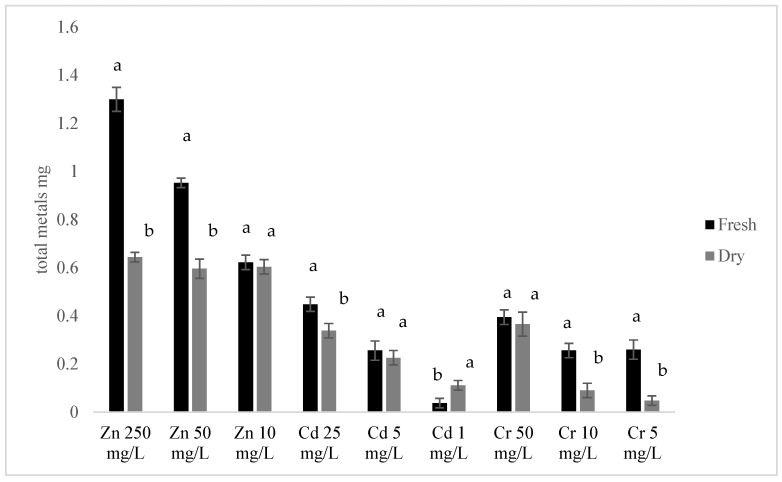
Total metals (mg) sequestered in fresh or dry duckweed from different metal solutions after 168 h. Different letters indicated a significant difference between dry and fresh duckweed at *p* < 0.05. n = 4.

The total metal sequestered in fresh and dried duckweed following a 168 h exposure period is presented in Figure 4. Duckweed accumulated significantly higher total quantities of metals when grown in high-concentration solutions (50 mg/L Cr^6+^ + 25 mg/L Cd^2+^ + 250 mg/L Zn^2+^). For example, after 168 h, fresh duckweed accumulated 0.39 ± 0.03 mg Cr^6+^, 0.44 ± 0.03 mg Cd^2+^, and 1.30 ± 0.05 mg Zn^2+^. These amounts were substantially greater than the 0.26 ± 0.03 mg Cr^6+^, 0.26 ± 0.04 mg Cd^2+^ and 0.95 ± 0.02 mg Zn^2+^ accumulated in middle-concentration solutions (10 mg/L Cr^6+^ + 5 mg/L Cd^2+^ + 50 mg/L Zn^2+^) or low-level metal solutions (5 mg/L Cr^6+^ + 1 mg/L Cd^2+^ + 10 mg/L Zn^2+^).

While fresh duckweed demonstrated a higher overall metal sequestration capacity over the 168 h trial, the efficacy of dried duckweed was comparable for certain situations. For instance, in low-level Zn^2+^ solutions (10 mg/L Zn^2+^) or middle-level Cd^2+^ (5 mg/L Cd^2+^), both forms accumulated similar amounts of Zn^2+^ and Cd^2+^ (Figure 4). Interestingly, dried duckweed even sequestered substantially more (0.11 ± 0.02 mg Cd^2+^) than fresh duckweed (0.04 ± 0.02 mg Cd^2+^) from low-level Cd^2+^ solutions (1 mg/L Cd^2+^).

**Figure 5:**
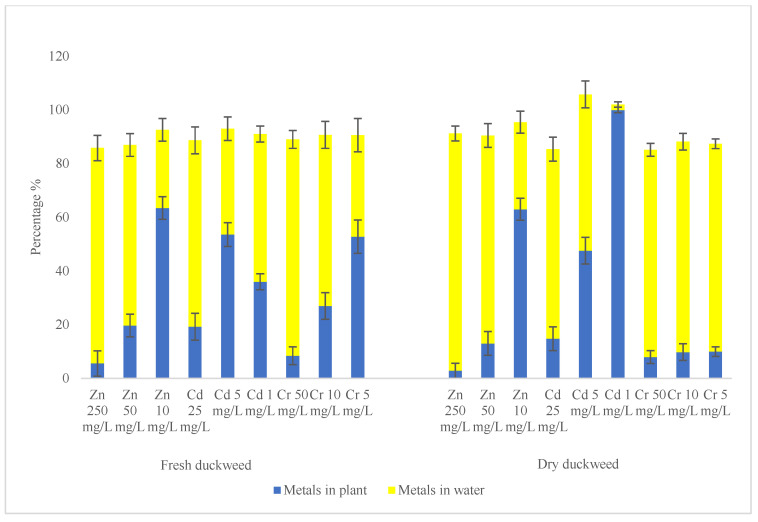
The percentages of metals sequestered in duckweed and remaining in water after 168 h of exposure.

The percentages of metals sequestered in fresh and dried duckweed, along with the amounts remaining in water after a 168 h exposure, are presented in Figure 5. While duckweed accumulated more metals when exposed to solutions with elevated metal concentrations (Figure 4), the corresponding removal rates were comparatively lower (Figure 5). For example, only 5.59 ± 0.10% of Zn^2+^ was accumulated in fresh duckweed and 2.86 ± 0.08% in dried duckweed from high-metal solutions (50 mg/L Cr^6+^ + 25 mg/L Cd^2+^ + 250 mg/L Zn^2+^), whereas more than 60% of Zn^2+^ was sequestered in both fresh and dried duckweed from low-metal solutions (5 mg/L Cr^6+^ + 1 mg/L Cd^2+^ + 10 mg/L Zn^2+^). Similarly, approximately 8% of Cr^6+^ was sequestered in both fresh and dried duckweed from high Cr^6+^-solutions (50 mg/L Cr^6+^), while more than 50% of Cr^6+^ was removed by fresh duckweed from low-Cr solutions (5 mg/L Cr^6+^). This trend was expected, as removal efficiency depends not only on the amount of metals taken up by plants but also on the initial metal concentrations in the solution: higher concentrations may result in greater uptake in terms of mass, but a lower percentage of removal [36]. Nevertheless, the steady accumulation of metals in fresh duckweed and the rapid sequestration in dried duckweed highlighted the strong potential of duckweed for metal removal.

## 3. Discussion

### 3.1. Phytoremediation by Fresh Duckweed

Phytoremediation experiments demonstrated that fresh duckweed steadily and consistently accumulated Cr^6+^, Cd^2+^ and Zn^2+^ among different concentrations over the 168 h period. This finding was in agreement with previous research. For example, Ajiboye et al. [37] confirmed the ability of *L. minor* to significantly remove Cr^6+^, Cd^2+^, and Zn^2+^ from water. Hasaballah [38] also indicated high sequestration of Cd^2+^ and Zn^2+^ in *L. minor*. In addition, Al-Nabhan [39] evaluated the removal efficiency of Cd^2+^, Pb^2+^, and Cr^6+^ (1–8 mg/L) by *L. minor* over 12 days and found the sequestration values of 3.2 mg/g dry biomass for Cd^2+^, 4.8 mg/g dry biomass for Pb^2+^, and 4.0 mg/g dry biomass for Cr^6+^ in duckweed—values higher than those observed in our study. Bokhari et al. [40] used another duckweed species, *Lemna gibba*, exposed to industrial effluent containing Ni^2+^ (2.15 mg/L), Pb^2+^ (1.51 mg/L), and Cd^2+^ (0.74 mg/L) over 21 days. They also reported significant metal removal efficiency, with accumulation values of 0.014 mg/g dry biomass for Cd^2+^, 0.051 mg/g dry biomass for Ni^2+^, and 0.036 mg/g dry biomass for Pb^2+^ in duckweed. Collectively, these studies demonstrated that duckweed had great potential as a candidate for phytoremediation, while also highlighting that metal accumulation in plants depends on factors such as the type and concentration of metals.

The steady accumulation of metals in our experiment also indicated a continuous dynamic interaction between dissolved metal ions and the plant’s metabolic and structural characteristics, which rely on active physiology beyond mere surface binding. Previous research also indicated that plant uptake of heavy metals from aqueous media is frequently characterized by a biphasic mechanism [41,42]. This consists of fast uptake due to adsorption and ion exchange on functional groups of cell wall constituents and other extracellular polymeric substances, followed by slower but progressive uptake by active transport across membranes and sequestration within tissues [41,42]. Our results demonstrated a sustained uptake phase over 168 h, indicative of active physiological processes dominating after the saturation of surface sites. This biphasic pattern—an initial rapid adsorption followed by prolonged internalization—is consistent with that reported for other aquatic macrophytes, including *L. minor* and *Eichhornia crassipes*, for metals like copper, nickel, zinc, and lead [41,43,44,45]. The observed continuation of uptake was driven by active metabolic processes and internal detoxification pathways within the plant cells [43,44,45]. In addition, the rhizosphere of plants plays an important role in the success of phytoremediation. The plant–root interface is a dynamic zone where physical, chemical, and biological interactions determine the fate of pollutants [46]. It also helps the plant to be more tolerant to metals and steadily accumulate them through mechanisms such as the release of chelating agents like organic acids to transform highly toxic metal species into less harmful forms, and the production of plant growth-promoting substances [47]. These processes help transform a simple plant into a powerful, resilient system for pollutant cleanup.

The Michaelis–Menten model is widely applied in phytoremediation research to describe metal uptake by plants, including essential micronutrients, such as Cu^2+^, Fe^2+^, and Mn^2+^ [48,49], as well as toxic metals such as Cd^2+^ [50]. For example, Cd^2+^ accumulation in seagrass tissues was effectively described by a Michaelis–Menten model [41,51]. In our study, Michaelis–Menten kinetics also provided a good fit for Cd^2+^ and Zn^2+^ uptake but not for Cr^6+^. This was not surprising, as the applicability of Michaelis–Menten equations may be related to the toxicity and transformation pathways of metals [26,52]. The transport pathways for Cr^6+^ are likely different from those for Cd^2+^ and Zn^2+^, as Cr^6+^ undergoes multiple transformations within biological systems, including reduction to Cr^3+^ [26]. These more complicated steps may prevent the Cr^6+^ uptake from conforming to Michaelis–Menten kinetics, which is different from Cd^2+^ and Zn^2+^. Previous research also reported that Michaelis–Menten uptake kinetics fit Zn^2+^ uptake in many plants, such as *Arabidopsis* and sunflower [53,54]. Ali et al. [22] indicated that Cr^6+^ uptake in plants occurs via non-specific channels or transporters, and its competitive interactions with essential ions result in Cr^6+^ influx not fitting a Michaelis–Menten model.

In the Michaelis–Menten model, the half-saturation constant (K_m_) reflects the affinity of plant transport systems for a given metal, while the maximum uptake rate (V_max_) represents the plant’s physiological capacity for metal transport [49]. In this study, V_max_ for Zn^2+^ and Cd^2+^ exceeded 20 mg/(g·h), although few previous studies have reported V_max_ values for these metals. For comparison, ammonium uptake in *L. minor* was estimated at 0.082 mg/(g·h) with a K_m_ of 1.88 mg/L [55], while arsenate uptake in *Spirodela polyrhiza* reached 0.47 ± 0.02 mg/(g·h) [56]. The K_m_ values observed in this study (~0.1 mg/L for Cd^2+^ and Zn^2+^) differ from those reported for other species, such as 0.6 mg/L for Zn^2+^ in *Odontarrhena chalcidica* [57] and 0.05 mg/L for Cd^2+^ in *Thlaspi caerulescens* [58]. Such variation is expected, as K_m_ is an apparent parameter reflecting the affinity of the uptake system under specific experimental conditions and can be influenced by factors including plant species, root surface characteristics, transporter expression levels, and solution chemistry [59].

The Michaelis–Menten model links zero-order and first-order kinetics. When metal concentrations in the environment are much lower than K_m_ [60], uptake follows first-order kinetics. At sufficiently high external concentrations, however, metal uptake by plants becomes independent of concentration, exhibiting zero-order kinetics [49]. In our study, the concentrations of metals in the solutions were all higher than K_m_. Thus, it is not surprising that the accumulations of Cr^6+^, Cd^2+^, and Zn^2+^ in duckweed also followed zero-order kinetics rather than first-order kinetics. These findings align with previous studies, which also reported that metal accumulation in plants when bioavailable metal was present in wastewater exhibited zero-order kinetics [25,26,27]. Zero-order metal uptake in phytoremediation reflected that the plant absorbed metals at a constant rate determined by its physiological limits (e.g., transporter abundance and metabolic activity) rather than by the metal concentration in the external environment, whereas accumulation rates in a first-order process decrease as concentration gradients diminish [48,49]. Zero-order kinetics further indicated that duckweed’s uptake process was not diffusion-limited but driven by active physiological transport. It also implied that metal accumulation in plant tissues increased linearly with time. The significant differences in metal accumulation between successive sampling times in this study further reflected that uptake was dynamic and sustained throughout the seven-day exposure period. This temporal pattern suggested that duckweed maintained an effective metal translocation and sequestration system and possessed substantial, largely unsaturated intracellular storage capacity—likely involving vacuolar compartmentalization and metal–chelator complexes—allowing continuous accumulation without early saturation or inhibition during the experiment [61]. This contrasts sharply with the rapid plateau observed in non-living biosorbents, where adsorption ceases once surface functional groups are saturated [25]. In living duckweed, metabolic activity enabled transport of the metals into the cytoplasm and sequestration into vacuoles, which released surface sites for further adsorption. Thus Cr^6+^, Cd^2+^ and Zn^2+^ accumulation increased with time in this study. Oporto et al. [41] observed similar phenomena for *L. minor* in constructed wetlands, with Cr^6+^ uptake continuing over several days without decline, supporting the interpretation that the ability of duckweed to utilize intracellular storage was key to its sustained accumulation. Similarly, Chua et al. [24] found that the kinetics of copper removal by bamboo species were best described by a zero-order model in an 80μM solution, yielding a strong fit (R^2^ = 0.95).

In addition to zero-order kinetics and the Michaelis–Menten model, first-order and pseudo-second-order kinetic models were reported in other phytoremediation experiments. For example, the uptake and transformation of the pharmaceutical metformin in cattails (*Typha latifolia*) over a 28-day experiment were best described by first-order kinetics [62]. In addition, kinetic order has also been observed to change under Cd^2+^ and Zn^2+^ stress conditions in other duckweed studies [42,44]. The choice of the best equation depends on several factors, including the medium conditions, plant species, and the concentration and form of the metal [25,26].

Duckweed accumulated different amounts of Cr^6+^, Cd^2+^ and Zn^2+^ into the tissues. This was due to different concentrations and toxicity of those metals. Plants also have different mechanisms to deal with different types of metals. As an essential element, Zn^2+^ was often complexed by phytochelatins and metallothioneins [44,63]. These peptides and proteins were induced by heavy metal stress and involved in chelating and detoxifying the metal ions in the cells [44,63]. The continuous synthesis of these peptides and proteins provided new binding capacity in the cytoplasm, thus ensuring uptake over a long time. Cr^6+^ is not an essential element, but it can be reduced from Cr^6+^ to the less toxic Cr^3+^ within plant tissues and subsequently stabilized through binding to organic ligands in aquatic macrophytes like duckweed and water hyacinth [45]. This conversion reaction, alongside other detoxification mechanisms like vacuolar compartmentation [42], enhanced long-term Cr^6+^ bioaccumulation in duckweed.

In addition, the amounts of Cr^6+^ and Cd^2+^ in fresh duckweed were much lower than those of Zn^2+^, which may be due to the higher concentrations of Zn^2+^ in the solutions. Previous research also indicated that metal accumulation in plants is positively related to metal concentrations in the growth medium [64]. Higher levels of Zn^2+^ in plants and the environment may also inhibit the uptake of other elements into plant tissues. As indicated by previous studies [65], elevated levels of one metal can inhibit the uptake and accumulation of other metals in plant tissues due to competition for shared uptake and transport systems. For example, Cd^2+^ and Zn^2+^ have very similar chemical properties and compete for the same binding sites on plant roots, entering the plant via the same transporters (e.g., ZIP family) [66]. Consequently, the presence of one metal can inhibit the uptake of the other. Furthermore, all metals must be loaded into the xylem to reach aboveground tissues, so high levels of one metal may create a bottleneck that blocks the transport of other metals into the biomass [67].

### 3.2. Biosorption by Dry Duckweed

Based on the results of biosorption experiments, Cr^6+^, Cd^2+^ and Zn^2+^ had different equilibrium times; the kinetic data were best described by the pseudo-second-order model; and the adsorption isotherm data fit the Langmuir model better than the Freundlich model. Upatham et al. [32] investigated the biosorption of Cd^2+^ and Cr^6+^ in duckweed *Wolffia globosa* and also confirmed that the adsorption equilibria followed Langmuir models. Similar to other macrophyte biosorbents [25,68,69], dried duckweed exhibited key trends of varying equilibrium times, chemisorption-based kinetics, and monolayer adsorption. These mechanisms, governed by ion chemistry and biomass structure, directly supported its practical use in batch adsorption systems and as a polishing agent in constructed wetlands [41,45].

Zn^2+^ reached equilibrium the fastest (4 h), Cd^2+^ took 4–24 h, and Cr^6+^ required up to 48 h. This variation was attributed to the intrinsic characteristics of each ion, specifically its hydration radius, charge density, and affinity for functional groups on the biomass surface [26,42]. Zn^2+^, a small divalent cation, diffused rapidly and interacted readily for reactive surface groups (e.g., hydroxyl, carbonyl, and amino), leading to its rapid and efficient removal. This finding aligns with prior biosorption studies on *L. minor* and *E. crassipes* [25,68]. In contrast, the larger ionic radius of Cd^2+^ and lower hydration energy slowed its diffusion to binding sites. Although Cd^2+^ had a high affinity for functional groups (particularly carbonyl and hydroxyl), its larger size and competition with background divalent cations like Ca^2+^ and Zn^2+^ prolonged its equilibrium time [26,69]. Cr^6+^ had the slowest adsorption kinetics, which was likely due to its complex aqueous chemistry. In contrast to the divalent cations, Cr^6+^ is present as oxyanions and must be partially dehydrated, hydrolyzed, or reduced to form stable complexes with biomass functional groups [26,69]. This necessary pre-adsorption step acts as a kinetic barrier, significantly reducing the rate of interaction with binding sites. This mechanism is supported by evidence from previous laboratory adsorption experiments and pilot-scale constructed wetland studies [41,45]. Consequently, these findings confirm that the adsorption equilibrium is not a universal constant but is intrinsically dependent on the specific chemical interplay between metal ions and functional groups.

The adsorption kinetics were best described by the pseudo-second-order model, with high correlation coefficients (R^2^ > 0.95) across all treatments. This strong fit indicated that chemisorption—a process involving the formation of covalent bonds via electron sharing or ion exchange—was the primary mechanism controlling metal sorption, rather than physisorption or diffusion [68,70]. The prevalence of chemisorption was attributed to the abundance of hydroxyl, carboxyl, and amino functional groups on the duckweed biomass, which provide ample sites for these valence-driven interactions [26,42]. This finding was consistent with biosorption trends observed across a broad array of macrophytes [69]. Specifically, prior studies on *L. minor*, *L. punctata*, *E. crassipes* and *S. polyrhiza* also reported pseudo-second-order kinetics for heavy metal removal, identifying hydroxyl and carbonyl groups as key binding sites [25,26,71,72]. The rapid initial uptake of all three metals also further supported a chemisorption mechanism. This indicated that binding sites were immediately accessible and that the rate-determining step was the chemical reaction itself, not diffusion. Consequently, unlike in living tissues, the active transport mechanisms present in fresh duckweed were absent, confirming that binding in the dried biomass was governed solely by surface chemistry [41].

The Langmuir isotherm model (R^2^ > 0.99) fit better than the Freundlich model (R^2^ = 0.83–0.94) for all three metals (Table 3). This indicated that adsorption occurred as a monolayer on a homogeneous surface with a finite number of identical binding sites, rather than through multilayer adsorption on a heterogeneous surface. The high correlation with the Langmuir model implied that the dried duckweed biomass possessed a relatively uniform distribution of functional groups. The clear plateau in adsorption at equilibrium, a hallmark of Langmuir behavior, signifies that Q_m_ was reached with no potential for further multilayer uptake (Table 3). This was further evidenced by Zn^2+^ biosorption, where the sorption capacity of dried duckweed plateaued at approximately 3.9 mg/g dry biomass despite increasing Zn^2+^ concentrations in the solution (Table 3; Figure 2). The maximum adsorption capacities observed in our study for dried duckweed (Cr^6+^: 8.96, Cd^2+^: 1.90, Zn^2+^: 3.90 mg/g dry biomass) were lower than many values reported in the literature. For instance, other studies have reported capacities of more than 30 mg/g dry biomass for Cd^2+^ in *Lemna aequinoctialis* and in *Spirodela polyrhiza* [25,73]. Using initial concentrations of 10–400 mg/L, Upatham et al. [32] reported Q_m_ values of 80.7 mg/g dry biomass for Cd^2+^ and 73.5 mg/g dry biomass for Cr^6+^ in dried duckweed *Wolffia globosa*. This discrepancy is likely due to the use of a small amount of biomass, different initial concentrations and the varying affinities of different metals [32]. In addition, previous research also indicated that although Q_m_ values typically decreased in multi-metal systems due to competition for binding sites, the Langmuir model remains valid and widely applied for determining adsorption capacities in mixed-metal solutions [74,75,76]. Thus, although the Q_m_ values in our study were not directly comparable to those from optimized systems, our data fitted the Langmuir model and provided a realistic assessment of dry duckweed’s performance under moderate, environmentally relevant conditions.

This site-limited, monolayer process is consistent with findings for other macrophyte biosorbents. For instance, Langmuir-type adsorption has been reported for Pb^2+^ uptake on duckweed and for Ca^2+^ and Zn^2+^ on *L. minor*, confirming that site-limited processes govern removal [25]. Similar behavior in *E. crassipes* (water hyacinth) and *Pistias tratiotes* has also been attributed to homogenous binding surfaces provided by lignocellulosic cell walls [71,72,77]. For instance, extensive research on *E. crassipes* has demonstrated pseudo-second-order kinetics and Langmuir isotherm fits for metals including Pb^2+^, Cr^6+^ and Cd^2+^ [71]. The primary functional groups responsible for binding in water hyacinth—hydroxyl and carboxyl moieties from cell wall polysaccharides—are identical to those identified in duckweed. Similarly, *P. stratiotes* (water lettuce) exhibited Langmuir-type adsorption for Zn^2+^ and Cd^2+^, reaching equilibrium within hours in a concentration-dependent manner, much like the behavior observed in this study [78]. The convergence of these mechanisms across diverse species reinforced that chemisorption and monolayer, site-limited binding were fundamental characteristics of macrophyte biosorption [68]; While duckweed shares this general sorptive behavior with other plants, it offered distinct practical advantages due to its small size, rapid growth rate, and ease of harvest, underscoring its strong potential as a biosorbent [44].

### 3.3. Comparison of Fresh and Dry Duckweed

This comparison elucidated the distinct metal accumulation patterns between fresh and dried duckweed. The consistent hierarchy of metal accumulation (Zn^2+^ > Cr^6+^/Cd^2+^) observed in fresh biomass reflected fundamental biological mechanisms: the essentiality and specialized transport systems of Zn^2+^ facilitated its preferential uptake and Cr^6+^ accumulation depends on speciation and competition for shared transport pathways, while cellular toxicity defenses significantly constrain Cd^2+^ accumulation [26,79]. In dried biomass, early time metal removal was governed by physicochemical factors such as site affinity and hydration radii. Cd^2+^, with its high affinity for oxygen-donor functional groups, exhibited particularly rapid initial adsorption until site saturation occurred. This behavior is supported by the high rate of cadmium removal at low concentrations observed in this study (Figure 3) and is consistent with the broader duckweed biosorption literature [25,80].

The collective evidence from these comparative studies indicated that fresh duckweed generally achieved a higher total uptake of Zn^2+^, Cr^6+^ and Cd^2+^ than its dried tissues—a trend best explained by the fundamental distinction between living and non-living biomass. Fresh duckweed exhibited sustained uptake over 168 h, indicative of active metabolic processes and intracellular storage [43]. In contrast, dried duckweed reached equilibrium rapidly, within hours, through adsorption controlled solely by surface functional groups and chemisorption [25]. This mechanistic difference directly influenced performance: fresh duckweed exhibited increased metal accumulation for over 168 h, while dried biomass reached a saturation equilibrium within hours, once its finite binding sites were occupied [26,43]. These findings align with the broader literature distinguishing active phytoremediation from passive biosorption in macrophytes. While fresh duckweed accumulated metals over longer periods and adapted to external conditions, dried duckweed offered the advantage of immediate and predictable removal within a short timescale—a trend consistently observed in other macrophytes [26,72].

However, as mentioned earlier, dried duckweed sequestered more Cd^2+^ than fresh duckweed from low-level Cd^2+^ solutions. This may be because fresh duckweed required time to activate detoxification pathways (e.g., phytochelatins and antioxidant enzymes) when exposed to even low levels of Cd^2+^, which can still cause physiological stress [81]. In contrast, dried biomass, being unaffected by metal toxicity, allowed for immediate contact and binding, enabling rapid metal sequestration. Previous research also indicated that dried plant biomass, including duckweed, effectively removed heavy metals from dilute solutions through surface biosorption mechanisms, often outperforming living biomass at low metal concentrations [19,26]. Conversely, fresh duckweed can exhibit stronger metal uptake at higher concentrations due to active transport and intracellular sequestration processes characteristic of living plants [17].

Another critical issue to consider in phytoremediation is how to prevent secondary contamination of the environment from contaminated biomass. Two promising approaches for utilizing phytoremediation biomass are the production of bioenergy, which is more sustainable compared to fossil fuels, and the generation of hydrochars that can be used as fuels, sorbents, and catalysts [82]. Different types of bioenergy can be produced through various thermochemical treatments of biomass, including anaerobic digestion, pyrolysis, hydrothermal conversion, and gasification [83].

Similarly, the management of metal-laden biomass following biosorption is also a critical consideration for ensuring the sustainability of biosorption processes. Some strategies have been developed to address this challenge, focusing on biomass reuse, energy recovery, and metal recuperation depending on the type of biosorbent and the amount of biosorbed metal [84]. However, research related to the post-use processing of contaminated biosorption biomass remains very limited and requires further investigation.

In addition, although numerous studies have been conducted on phytoremediation and biosorption of metal-contaminated environments, most have been conducted at the laboratory scale with limited cases of commercialization [85,86]. For the application of phytoremediation and biosorption technologies on an industrial scale, economic analyses are required to obtain the overall cost of the process to treat large volumes of wastewater.

## 4. Materials and Methods

### 4.1. Plant Preparation

Fresh duckweed, purchased from Play It Koi (Bothell, WA, USA), was cleaned with distilled water and acclimated for one week in a distilled water solution containing 2 g/L of commercial MaxiGro nutrients (MiracleGro company, Marysville, OH, USA), which comprised 10.0% total nitrogen, 5.0% available phosphate (P_2_O_5_), 14.0% soluble potassium (K_2_O), 6.0% calcium, 2.0% magnesium, 3.0% sulfur, 0.12% iron, 0.05% manganese, and 0.002% molybdenum. The acclimated plants were then split for two purposes: one portion was used directly in phytoremediation experiments, while the other was then dried at 60 °C for metal adsorption experiments [87].

### 4.2. Metal Solutions

The control solutions contained 0.2 g of commercial MaxiGro nutrients per 100 mL of deionized water. The metal solutions were prepared with the same nutrient base and spiked with three target metals (Cr^6+^, Cd^2+^, and Zn^2+^), using their analytical-grade salts, K_2_Cr_2_O_7_, CdCl_2_, and ZnCl_2._ All the Chemicals were purchased from company “Fisher Scientific” (Portsmouth, NH, USA) online). These metals were added to create three different concentration levels, which were selected based on the metal concentrations typically found in industrial wastewater, as reported in prior studies [88,89]: low (5 mg/L Cr^6+^, 1 mg/L Cd^2+^, and 10 mg/L Zn^2+^), middle (10 mg/L Cr^6+^, 5 mg/L Cd^2+^, and 50 mg/L Zn^2+^), and high (50 mg/L Cr^6+^, 25 mg/L Cd^2+^, and 250 mg/L Zn^2+^). Prior to the experiment, the pH of all solutions was adjusted to 6.5 using hydrochloric acid (HCl) or sodium hydroxide (NaOH). This pH value was chosen because it is common in wastewater and directly influences plant development while also affecting the efficacy of metal removal through biosorption [88,90].

### 4.3. Phytoremediation and Biosorption Experiment

For the phytoremediation experiments, acclimated duckweed was collected from nutrient solutions and rinsed thoroughly with distilled water. To remove surface moisture without causing stress to the living plants, the duckweed was placed on filter paper until excess surface water was removed. Approximately 4 g of fresh duckweed was subsequently transferred into 100 mL of either control or metal solutions under growing light (8 h per day) at room temperature. Samples were subsequently collected at 8, 24, 72, 120, and 168 h for analysis.

For the biosorption assay, dried biomass equivalent to approximately 4 g of fresh duckweed (around 0.2 g dry weight) was ground to pass through a 2 mm sieve and subsequently added to 100 mL of control or metal solutions. The flasks were agitated constantly at 140 rpm at room temperature, and samples were collected at 2, 4, 8, 24, 48, and 168 h.

All duckweed samples from the phytoremediation and biosorption experiments were first oven-dried at 60 °C until a constant weight was reached and then weighed [87] prior to acid digestion [91,92]. The acid digestion method was based on previous research and the EPA hot-plate acid digestion protocol [39,93]. Briefly, 1 g or less of plant tissue was soaked in 20 mL of 70% nitric acid for 6 h. The mixture was then boiled down to 10 mL, after which 4 mL of 70% perchloric acid was added and refluxed for 90 min. Finally, the solution was diluted to 20 mL with distilled water. The recovery rate of this method can exceed 80% [94,95]. The resulting digestates were preserved at 4 °C in a refrigerator and then analyzed by Inductively Coupled Plasma–Optical Emission Spectroscopy ICP-OES (Thermo Scientific iCAP PRO Series ICP-OES system from Company “Thermo Fisher Scientific” in Waltham, MA, USA).

### 4.4. Kinetic and Isotherm Models

#### 4.4.1. Metal Uptake Kinetics by Fresh Duckweed

The metal uptake kinetics in fresh duckweed were related to the total metals in duckweed over a certain time. The main kinetic models for metal accumulations for phytoremediation included zero-order, first-order, and Michaelis–Menten models [24].

The zero-order kinetic model is described by the following equation [24]:

$$C_{L}=k_{0}t$$

where C_L_ is the amount of metals sequestered in per mass of the plants (mg/g dry biomass) at time t(h) and k_0_ is the zero-order rate constant.

The first-order kinetic model of metal uptake in plants can be illustrated by the following equation [24]:

$$ln\left[\frac{\frac{M_{i}}{w}-C_{L}}{\frac{M_{i}}{w}}\right]=k_{1}\times ln\left[\frac{\frac{V_{i}}{w}-(at+b)}{\frac{V_{i}}{w}-b}\right]$$

where C_L_ is also the mass of metals sequestered from the media into per dry mass of the plant, V_i_ is the initial volume of the solution, M_i_ is the initial mass of metals in the media, w is the mass of the plant, and a and b are the slopes and intercept in plotting. k_1_ is the first-order rate constant.

The other model is the Michaelis–Menten equation [24]:

$$\frac{t}{C_{p}}=\frac{K_{M}}{V_{max}C_{i}}+\frac{1}{V_{max}}$$

where C_i_ is the initial concentrations of substrates (e.g., metals) in solutions, C_p_ also refers to the concentrations of metals in the plant, K_M_ is the Michaelis–Menten constant and V_max_ is the maximum reaction velocity, which can be reflected by plotting.

#### 4.4.2. Metal Biosorption Kinetics

The kinetics of metal biosorption onto dried duckweed biomass were analyzed using pseudo-first-order and pseudo-second-order models, which are widely used to describe solute adsorption dynamics [25].

The linear form of the pseudo-first-order model is given by the following equation [26]:

$$\ln(Q_{e-}Q_{t})=ln(Q_{e})-k_{1}t$$

where Q_e_ and Q_t_ are the amounts of metal adsorbed (mg/g dry biomass) at equilibrium and at time t, respectively, and k_1_ is the pseudo-first-order rate constant (time^−1^). The pseudo-first-order kinetic parameters were determined using Microsoft Excel by plotting ln (Q_e_-Q_t_) versus t. The rate constant k_1_ and the equilibrium capacity Q_e_ can be determined from the slope and the intercept of the plot, respectively [26,96]

The linear form of the pseudo-second-order model is expressed as follows [96]:

$$\frac{t}{Q_{t}}=\frac{1}{k_{2}{Q_{e}}^{2}}+\frac{t}{Q_{e}}$$

where k_2_ is the pseudo-second-order rate constant g/(mg·h). The constants of pseudo-second-order kinetics were defined by plotting t/Q_t_ versus t. The rate constant k_2_ and the equilibrium capacity Q_e_ can be calculated from the intercept and the slope of the plot, respectively [96]. These models were fitted to the experimental data to identify which one best describes the biosorption mechanism.

#### 4.4.3. Adsorption Isotherms

Adsorption isotherms characterize the distribution of metal ions between the liquid phase and the solid adsorbent at equilibrium. Two common isotherm models, the Langmuir and Freundlich, were employed to analyze the biosorption data for dried duckweed [96,97].

The Langmuir isotherm model, which assumes monolayer adsorption onto a surface with a finite number of identical sites, was expressed as follows [96]:

$$\frac{C_{e}}{Q_{e}}=\frac{1}{Q_{m}K_{L}}+\frac{C_{e}}{Q_{m}}$$

where Q_e_ is the amount of metal adsorbed per unit mass of biomass at equilibrium (mg/g dry biomass), C_e_ is the equilibrium metal concentration in the solution (mg/L), Q_m_ is the maximum adsorption capacity (mg/g dry biomass), and K_L_ is the Langmuir constant (L/mg). A plot of C_e_/Q_e_ versus C_e_ yields a straight line, with the slope equal to 1/Q_m_ and the intercept equal to 1/(Q_m_K_L_) [96,98].

The Freundlich isotherm model, which describes multilayer adsorption on a heterogeneous surface, was applied in its linear form [96,97]

$$\log(Q_{e})=\log\left(K_{F}\right)+(\frac{1}{n})\log\left(C_{e}\right)$$

where *K_F_* is the Freundlich constant, and n is a dimensionless constant related to the adsorption intensity.

The fit of these models was evaluated to determine which one better describes the equilibrium biosorption of metals onto dried duckweed.

### 4.5. Statistical Analysis

All the experiments were conducted with four replicates. All data were checked for normality and homogeneity of variances. Then, statistics analysis was conducted utilizing one-way ANOVA in MINITAB (version 16). Tukey’s test was utilized at a 5% confidence level to identify significant differences among treatments.

## 5. Conclusions

In this study, metal uptake by fresh duckweed followed zero-order kinetics for Cr^6+^, Cd^2+^, and Zn^2+^ sequestration, while Cd^2+^ and Zn^2+^ uptake also conformed to Michaelis–Menten kinetics, rather than a first-order model. Biosorption using dried duckweed exhibited pseudo-second-order kinetics, with Zn^2+^ reaching equilibrium the fastest (4 h), followed by Cd^2+^ (4–24 h) and Cr^6+^ (up to 48 h). The adsorption isotherm was best described by the Langmuir model.

Fresh duckweed demonstrated a higher total metal sequestration capacity over the 168 h experiment. However, in low-level Zn^2+^ (10 mg/L) or medium-level Cd^2+^ (5 mg/L) solutions, both fresh and dried forms accumulated similar amounts of metal. In low-level Cd^2+^ (1 mg/L) solutions, dried duckweed even sequestered significantly more Cd^2+^ than fresh duckweed.

Dried duckweed acts as an efficient adsorbent, suitable for short-contact-time, high-rate applications targeting peak metal concentrations—especially for metals like Zn^2+^, which reached equilibrium the fastest. Its predictable Langmuir capacity and tolerance to toxic conditions make it ideal for primary treatment stages. In contrast, metal removal by fresh duckweed relies on metabolically driven uptake and intracellular storage over extended periods, with performance dependent on biomass productivity, nutrient status, and environmental conditions.

While dried biomass offers superior efficiency for rapid metal removal under optimized conditions, fresh biomass provides greater total capacity over longer durations. This functional complementarity enables treatment strategies in the real world to be arranged by selecting fresh biomass, dried biomass, or a combined approach, depending on specific operational needs such as water chemistry, contact time, and the desired balance between rapid adsorption and long-term accumulation. For example, a sequential treatment strategy can be implemented in which dried duckweed biomass is first applied in an adsorption unit for rapid bulk metal removal, followed by fresh duckweed pond to polish residual metal concentrations through phytoremediation. This intergraded approach combines the fast adsorption kinetics of dried duckweed biomass with the biological uptake capacity of living duckweed, which may maximize overall removal efficiency. Additionally, further studies are needed to address the sustainable management of metal-sequestered biomass from phytoremediation and biosorption to prevent secondary contamination, and to optimize these processes for economically feasible industrial-scale implementation.

## Figures and Tables

**Table 1:** a: R^2^ of the zero-order and first-order equations for metal accumulation in fresh duckweed. b: R^2^ of the Michaelis–Menten equation for metal accumulation in fresh duckweed.

a							
Metals			Zero-order R^2^			First order R^2^	
50 mg/L Cr			0.94			−0.22	
10 mg/L Cr			0.96			−0.16	
5 mg/L Cr			0.81			0.18	
25 mg/L Cd			0.78			0.84	
5 mg/L Cd			0.97			0.94	
1 mg/L Cd			0.82			0.71	
250 mg/L Zn			0.71			0.46	
50 mg/L Zn			0.76			0.98	
10 mg/L Zn			0.83			0.42	
b							
Metals	8 h	24 h	72 h	120 h	168 h	Avg K_m_	Avg V_max_
	R^2^	R^2^	R^2^	R^2^	R^2^	mg/L	mg/(g·h)
Cr	0.01	0.01	0.09	0.17	0.73	0.03	1.66
Cd	0.97	1	1	0.99	0.99	0.09	23.9
Zn	0.96	0.91	0.98	0.96	0.97	0.08	22.5

**Table 2:** Comparison of 1st- and 2nd-order kinetic of metal adsorption by dry duckweed.

Metals	1st-Order R^2^	K_1_	Q_e1_	2nd-Order R^2^	K_2_	Q_e2_	Q_e-exp_
		(h^−1^)	mg/g Dry Biomass		g/(mg·h)	mg/g Dry Biomass	
50 mg/L Cr	0.85	0.08	0.69	0.98	0.02	2.26	2.00
10 mg/L Cr	0.94	0.06	0.26	0.96	0.05	0.58	0.50
5 mg/L Cr	0.57	0.02	0.29	0.99	0.23	0.27	0.25
25 mg/L Cd	0.77	0.02	0.16	1.00	0.38	1.89	1.85
5 mg/L Cd	0.86	0.01	0.13	1.00	0.80	1.24	1.22
1 mg/L Cd	0.17	0.01	0.46	1.00	2.65	0.58	0.65
250 mg/L Zn	0.28	0.02	0.39	1.00	4.11	3.59	3.80
50 mg/L Zn	0.09	0.01	0.17	1.00	6.84	3.24.	3.40
10 mg/L Zn	0.85	0.08	0.69	1.00	0.50	3.16.	3.00

**Table 3:** Comparison of Langmuir isotherms and Freundlich isotherms for metal adsorption by dry duckweed.

Metal	Langmuir Isotherms R^2^	Q_m_ (mg/g Dry Biomass)	K_L_ (L/mg)	Freundlich Isotherms R^2^
Cr	0.99	8.96	0.01	0.89
Cd	1.00	1.90	1.50	0.94
Zn	1.00	3.90	0.33	0.83

## Data Availability

The raw data supporting the conclusions of this article will be made available by the authors on request.
